# Attract them anyway: benefits of large, showy flowers in a highly autogamous, carnivorous plant species

**DOI:** 10.1093/aobpla/plw017

**Published:** 2016-03-14

**Authors:** A. Salces-Castellano, M. Paniw, R. Casimiro-Soriguer, F. Ojeda

**Affiliations:** 1 Departamento de Biología and IVAGRO, Universidad de Cádiz, Campus Río San Pedro, E-11510 Puerto Real, Spain; 2 Present address: IPNA-CSIC, C/Astrofísico Francisco Sánchez 3, 38206-La Laguna, Tenerife, Canary Islands, Spain

**Keywords:** Autogamous selfing, *Drosophyllum lusitanicum*, floral display, pollination biology, prey capture, pyrophyte, seed set

## Abstract

Reproductive biology of carnivorous plants has largely been studied on species that rely on insects as pollinators and prey, creating potential conflicts. Autogamous pollination, although present in some carnivorous species, has received less attention. In angiosperms, autogamous self-fertilization is expected to lead to a reduction in flower size, thereby reducing resource allocation to structures that attract pollinators. A notable exception is the carnivorous pyrophyte *Drosophyllum lusitanicum* (Drosophyllaceae), which has been described as an autogamous selfing species but produces large, yellow flowers. Using a flower removal and a pollination experiment, we assessed, respectively, whether large flowers in this species may serve as an attracting device to prey insects or whether previously reported high selfing rates for this species in peripheral populations may be lower in more central, less isolated populations. We found no differences between flower-removed plants and intact, flowering plants in numbers of prey insects trapped. We also found no indication of reduced potential for autogamous reproduction, in terms of either seed set or seed size. However, our results showed significant increases in seed set of bagged, hand-pollinated flowers and unbagged flowers exposed to insect visitation compared with bagged, non-manipulated flowers that could only self-pollinate autonomously. Considering that the key life-history strategy of this pyrophytic species is to maintain a viable seed bank, any increase in seed set through insect pollinator activity would increase plant fitness. This in turn would explain the maintenance of large, conspicuous flowers in a highly autogamous, carnivorous plant.

## Abstract (precis)

Despite being a highly autogamous or self-pollinating species, the carnivorous plant *Drosophyllum lusitanicum* (Drosophyllaceae) produces large, bright-yellow flowers. Our results detected a significant increase (15-25%) in seed set of experimentally self-pollinated flowers and flowers exposed to insect visitation compared with bagged, untouched flowers whose seeds were produced only by spontaneous self-pollination. Given that the key life-history stage of this fire-adapted plant species is the formation of a persistent seed-bank, any increase in seed production through insect pollinator activity would increase plant fitness. This in turn would explain the maintenance of large, showy flowers in a highly autogamous plant.

## Introduction

Carnivorous plants have long captivated naturalists and scientists worldwide (Chase *et al*. 2009; Król *et al*. 2012). Charles Darwin himself was most fascinated by them and was the first to demonstrate plant carnivory experimentally (Darwin 1875). Carnivory has evolved several times independently in the angiosperms and ∼600 species of carnivorous plants can be found today across the globe, most prominently in tropical and temperate regions (Heubl *et al*. 2006; Ellison and Gotelli 2009). They are largely restricted to infertile, wet, open habitats (Givnish *et al*. 1984) where they have adapted to extremely low nutrient levels by evolving elaborately modified leaves that trap small animals, mainly insects, as prey (Ellison and Gotelli 2001, 2009; Gibson and Waller 2009) and absorb the necessary mineral nutrients from them, particularly nitrogen and phosphorus (Adamec 1997).

Since most carnivorous plants are also entomophilous (i.e. they rely on pollinating insects to facilitate sexual reproduction), a pollinator–prey conflict might occur if they trapped potentially efficient pollinators (Zamora 1999; Ellison and Gotelli 2001). However, there are mechanisms in carnivorous plants to avoid or minimize this conflict, such as separation (spatial or temporal) of flowers from leaf traps to avoid pollinators being trapped as prey, or the occurrence of autogamous self-pollination to become somewhat independent of the role of insect vectors for reproduction (Ellison and Gotelli 2001; Jürgens *et al.* 2012). Autogamous self-pollination is actually common in some species from different carnivorous genera (see references in Jürgens *et al*. 2012).

**Figure 1.**
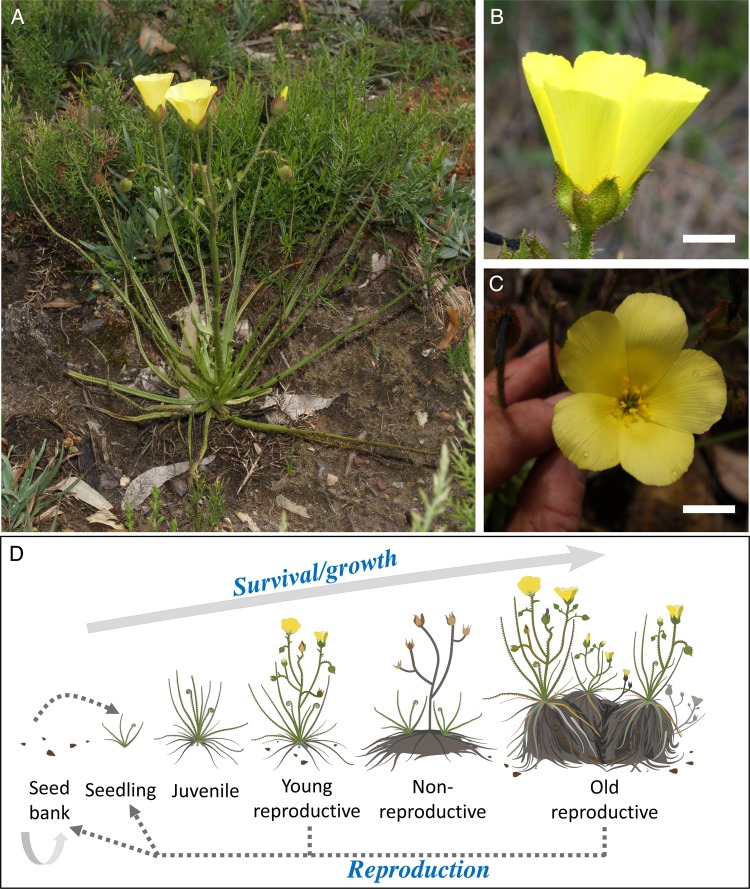
Visual description of *Drosophyllum*. (A) Young reproductive individual with a single rosette of leaves and a stalked inflorescences with two open flowers. (B) Lateral view of the flower showing the five large, bright yellow petals (scale bar = 10 mm). (C) Frontal view of the flower, showing the homogamous lack of separation between anthers and stigmas (scale bar = 10 mm). (D) Schematic description of the plant's life cycle.

*Drosophyllum lusitanicum* (Drosophyllaceae), the only extant species of the family Drosophyllaceae (Heubl *et al*. 2006), is an example of autogamous self-pollination in carnivorous plant species (Ortega-Olivencia *et al*. 1995, 1998). This species (*Drosophyllum*, hereafter) is endemic to the western Iberian Peninsula and northern Morocco (Garrido *et al*. 2003; Paniw *et al*. 2015), where it is restricted to acidic, nutrient-poor Mediterranean heathlands (Müller and Deil 2001; Adlassnig *et al*. 2006) and tightly associated to post-fire habitats (Correia and Freitas 2002; Paniw *et al*. 2015). *Drosophyllum* is a short-lived subshrub up to 45 cm tall with circinate, linear leaves grouped in dense rosettes and covered with stalked mucilage-producing glands (Paiva 1997). It produces large, sulfur-yellow, hermaphrodite flowers, radiate and pentamerous, borne in stalked, cymose inflorescences (Paiva 1997; Correia and Freitas 2002; Fig. 1). Flowers are homogamous, i.e. possess a spatial and temporal closeness between dehiscing anthers and receptive stigmas, with high selfing capability even in pre-anthesis (Ortega-Olivencia *et al*. 1995, 1998).

It is well established that autogamous selfing in angiosperms is favoured under pollinator limitation (Schemske and Lande 1985; Morgan and Wilson 2005), and it is usually accompanied by morphological changes in floral traits such as the occurrence of homogamy and a dramatic reduction in corolla size (Goodwillie *et al*. 2010; Sicard and Lenhard 2011). This reduction in flower size and other floral traits (e.g. showiness) is explained as a way to minimize resource allocation to floral display when pollinator attraction is no longer necessary (e.g. Andersson 2005; Celedón-Neghme *et al*. 2007). However, one of the noticeable features of the autogamous *Drosophyllum* is the production of large, showy flowers on peduncled inflorescences (Fig. 1). Therefore, considering the high allocation costs of flower production (Galen 1999; Andersson 2005), what are the benefits of large, conspicuous flowers in a carnivorous plant species presumably independent of the role of pollinating insects for reproduction (Ortega-Olivencia *et al*. 1995)?

Here, we present two field experiments on the floral and reproductive biology of *Drosophyllum* aimed to determine fitness benefits from the production of large, conspicuous flowers. First, assuming independence of pollinating insects for reproduction (Ortega-Olivencia *et al*. 1995), we explored whether the large, bright yellow corollas in this carnivorous species act as attracting devices for enhancing prey capture onto the sticky leaf traps, thereby supporting plant growth. Although there is virtually no overlap between prey and flower-visiting insect faunas (Bertol *et al*. 2015), it is well established that the bright yellow colour is attractive to many insect species, particularly flies (e.g. Neuenschwander 1982; Yee 2015), which are the most common prey in *Drosophyllum* (Bertol *et al*. 2015). Specifically, we hypothesized that flowering *Drosophyllum* plants whose flowers are removed would trap fewer prey insects than co-occurring, intact flowering plants, which would indicate an increase in plant fitness through insect capture resulting from maintenance of large, yellow flowers.

Second, we conducted a controlled pollination experiment to investigate the actual contribution of pollinators to fecundity (i.e. seed production) of this species. Unlike previous pollination experiments on this species (Ortega-Olivencia *et al*. 1995, 1998), which have been performed in geographically isolated, small populations, our experimental populations were located in the northern side of the Strait of Gibraltar, where populations are larger and more abundant (Garrido *et al*. 2003; Paniw *et al*. 2015). Since marginal populations of normally outcrossing plant species frequently show a considerable increase in the selfing rate (Lloyd 1980; Pujol *et al*. 2009), the highly autogamous self-fertilization of *Drosophyllum* reported previously might be contingent on geographical isolation. We predicted that attraction of pollinating insects by *Drosophyllum* flowers would increase fitness through an increase in fecundity in this carnivorous species, thus accounting for its large, conspicuous flowers.

## Methods

### Ecology of *Drosophyllum*

*Drosophyllum* is a disturbance-adapted, carnivorous species, colonizing (from a persistent seed bank) recently burned heathlands or heathland patches where small-scale disturbances create open space (Garrido *et al*. 2003; Paniw *et al*. 2015). Within 4–6 years after fire, regenerating heathland shrubs outcompete above-ground *Drosophyllum* individuals, making the formation of a seed bank—in which populations may persist for several decades until another fire—a critical life-history strategy (Paniw *et al*. 2015; M. Paniw, P. Quintana-Ascencio, F. Ojeda and R. Salguero-Gómez, unpublished). In habitats where small-scale disturbances, e.g. browsing, create and maintain open space, individuals may reach up to 10 years of age (Juniper *et al*. 1989). Individuals grow in rosettes, and number of rosettes is a good proxy for age. Plants 1–2 rosettes in size initially reproduce in the second year after emergence and the number of rosettes per plant increases each growing season (Ortega-Olivencia *et al*. 1995; Garrido *et al*. 2003; Fig. 1D). Demographic censuses of populations across southern Spain determined that each rosette produces one floral scape with an average (±SD) of 3.5 ± 2.1 flowers (M. Paniw, P. Quintana-Ascencio, F. Ojeda and R. Salguero-Gómez, unpublished). Bright sulfur-yellow flowers on each scape open gradually and last 1 day in full anthesis, so that no more than two flowers per rosette are in anthesis at the same time (Fig. 1). Flowers are large (Correia and Freitas 2002), with an average petal length of 2.84 ± 0.21 cm and petal width of 1.89 ± 0.17 cm (A. Salces-Castellano, unpubl. data), and show high autogamy rates (Ortega-Olivencia *et al*. 1995, 1998). High autogamy is also supported by the high inbreeding coefficients found in *Drosophyllum* populations (Paniw *et al*. 2014). Each flower produces a dehiscent capsule with an average of 9.8 ± 2.4 seeds (M. Paniw, P. Quintana-Ascencio, F. Ojeda and R. Salguero-Gómez, unpublished).

### Study region and sites

**Figure 2.**
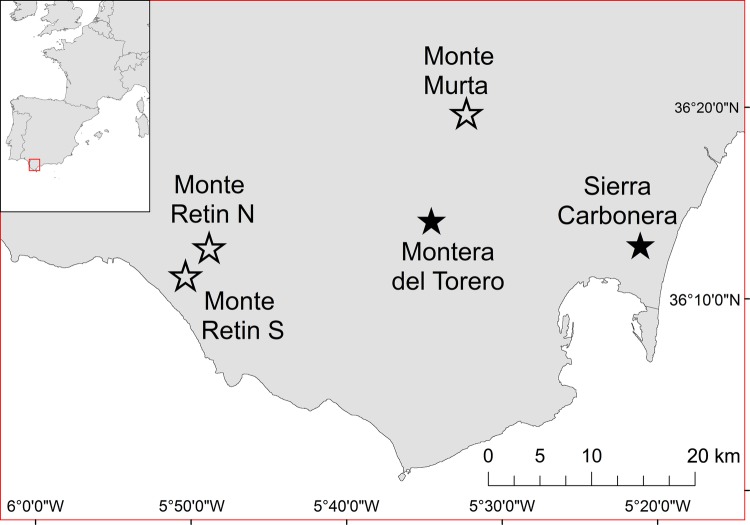
Study area and location of the sites where the flower contribution to prey attraction (open star) and pollination experiments (filled star) were performed. See Table 1 for detailed description of the *Drosophyllum* populations at each site.

Two field experiments were conducted in five natural *Drosophyllum* populations, located at five sites within the southern Aljibe Mountains, at the European side of the Strait of Gibraltar (Table 1; Fig. 2). From all its distribution range, this is where *Drosophyllum* is more abundant and populations are largest (Garrido *et al.* 2003; Paniw *et al.* 2015). This region is characterized by a mild Mediterranean climate (∼18 °C mean annual temperature and ∼1200 mm annual rainfall) and a rough topography dominated by Oligo-Miocene sandstone mountains and hills, which produce acidic, nutrient-poor soils in ridges and upper slopes (Ojeda *et al*. 2000). These infertile soils are covered by Mediterranean heathlands, dominated by dwarf shrubs like *Erica australis*, *Pterospartum tridentatum*, *Quercus lusitanica*, *Calluna vulgaris* and *Halimium lasianthum*, and are the primary habitat of *Drosophyllum* (Müller and Deil 2001; Paniw *et al*. 2015). Although this species is highly pyrophytic (i.e. associated with the recurrent presence of fire) and therefore threatened by large-scale anthropogenic activities such as afforestation (Andrés and Ojeda 2002) and fire suppression (Correia and Freitas 2002), it profits from small-scale vegetation clearances, where populations can still thrive (Garrido *et al*. 2003; Paniw *et al*. 2015).

**Table 1.** Description of sites used in the flower removal and pollination experiments quantifying the role of *Drosophyllum* flowers in prey capture and pollinator attraction, respectively. *N*, total number of *Drosophyllum* individuals found in 2014.

Site	Location	Experiment	Site characteristics	Population characteristics
Monte Murta	36°19′16″N 5°33′03″W	Flower removal	Open, rocky sandstone ridge	*N* = 5000; mixed-aged population
Monte Retin North	36°11′53″N 5°49′25″W	Flower removal	Open heathland patch	*N* = 1500; mixed-aged population
Monte Retin South	36°10′23″N 5°50′53″W	Flower removal	Post-fire regenerating heathland (fire 2010); browsed and trampled by cattle	*N* = 500; mixed-aged population
Sierra Carbonera	36°12′35″N 5°21′37″W	Pollination	Post-fire regenerating heathland (fire 2011)	*N* = 3000; mainly young reproductive individuals
Montera del Torero	36°13′35″N 5°35′08″W	Pollination	Mechanically built firebreak	*N* = 3700; mainly old reproductive individuals

We chose the study sites to represent the most common habitats of *Drosophyllum* populations (Paniw *et al*. 2015). Monte Murta is an open, rocky sandstone ridge with sparse heathland vegetation, which had been mechanically removed about 30 years ago for pine afforestation. In 2014, the *Drosophyllum* population consisted of ∼5000 individuals, where young flowering plants, consisting of 1–2 rosettes, and old flowering plants (>2 rosettes) co-occurred. Sierra Carbonera is a regenerating heathland patch from a fire suffered in early autumn 2011. The *Drosophyllum* population here was also large (∼3000 individuals) and consisted mainly of young flowering plants (2–3 years old), plus juveniles and a few seedlings. Montera del Torero is an old firebreak line across a heathland created by mechanical clearance of the vegetation. The *Drosophyllum* population at this site consisted of ∼3700 individuals and has persisted for >30 years, being dominated by old (>5 years) flowering plants. Lastly, two populations with different relative abundance of old reproductive individuals were encountered in Monte Retin. The population in Monte Retin North has persisted for >20 years in an open heathland on a rocky sandstone ridge. It consisted of ∼1500 individuals where old and young flowering individuals co-occurred. The population in Monte Retin South is found on a regenerating heathland patch from a fire suffered in early autumn 2010. This population, which has been heavily disturbed by cattle grazing and trampling, consisted of ∼500 individuals, with an even distribution of young and old reproductive individuals.

### Flower contribution to prey attraction

To test whether flowers in *Drosophyllum* functioned to attract prey insects, we carried out a field experiment at three of the five study sites, Monte Murta, Monte Retin North and Monte Retin South (Fig. 2) in April 2014, during peak flowering. At each site, we located ‘isolated’ flowering plants growing in open microhabitats (>1 m from the nearest conspecific and >30 cm from the nearest interspecific neighbour), in order to avoid potential influences of conspecific flowering neighbours on prey capture. We randomly marked 14 plants and recorded the number of rosettes and leaves per rosette of each plant. All prey insects were then carefully hand-removed with tweezers from each plant. Next, we randomly selected 7 plants out of those 14 and removed all their flowers by cutting off the inflorescence stalks with scissors. After 1 week, we returned to each of the three populations and recorded the number of prey insects attached to the leaves of the 14 plants.

We analysed the differences in insect capture between flower-removed plants (treatment) and intact ones (control) for each site separately by fitting a generalized linear model with a Poisson error distribution on the total number of insects, using the ‘flower-cut’ treatment as fixed effect and total number of leaves per plant as the offset. Using an offset allowed us to treat the response (number of insects) as proportions (insects per leaf) but allowing the models to be fit as count data in a generalized linear mixed model framework. The analyses were performed separately for each site because we did not have enough spatial replicates to include site as a random effect in our models (Bolker *et al*. 2009).

### Pollination experiment

We carried out an experiment at two of the five study sites, Sierra Carbonera and Montera del Torero (Fig. 2), to investigate the contribution of pollinators to *Drosophyllum* fecundity (i.e. seed production). In mid-April 2014, at the beginning of the flowering season, we labelled 56 and 43 plants in Sierra Carbonera and Montera del Torero, respectively. On each plant, flowers were randomly assigned to one of four treatments: hand cross-pollination (HCP), hand self-pollination (HSP), spontaneous self-pollination (SSP) and control or open pollination (OP). In the first three treatments, flowers were covered with nylon-mesh bags (0.15-mm mesh) before anthesis to exclude potential insect visitors. For the two hand-pollination treatments, HCP and HSP, we collected ripe anthers from plants separated >300 m (HCP) or from the same flower (HSP) and brushed the stigmas with them, taking care of bagging them back after this artificial pollination. Flowers in the SSP treatment were not hand-pollinated and remained bagged in order to account for spontaneous autogamy. Finally, flowers in the OP treatment (control) were left exposed to natural pollinator activity. In most plants, there was more than one flower for each treatment. We also collected a single petal from an extra flower per plant to measure petal length as a surrogate for flower size.

In July 2014, after fruit (capsule) ripening and before seed dispersal (dehiscence), we collected the fruits of the four treatments on each individual plant from the two sites. They were stored individually in labelled paper bags and taken to the laboratory, where we calculated fruit set (percentage of flowers within each treatment developing into fruits) and seed set (percentage of ovules per flower maturing into seeds) per treatment. Additionally, three randomly chosen seeds per fruit were weighed on an electronic balance to the nearest 0.1 mg and their length (as a surrogate for size) measured using an image analyser (Leica Application Suite v4.4.0, LAS v4.4, Leica Microsystems).

We tested for differences in fruit set, seed set, seed weight and seed size among pollination treatments by means of a mixed effect models with a binomial error distribution for the response variables fruit set and seed set and normal error distribution for the response variables seed weight and size. We considered treatment (OP, HCP, SCP and SSP) as fixed effect and plant individual as a random effect in all models. We fitted the models for each of the two sites separately.

All analyses were performed with R software (R Development Core Team 2015). We used the R package lme4 (Bates *et al*. 2013) to fit the mixed effect models. In both experiments described above, we used likelihood ratio tests to determine signiﬁcant differences between treatments (Vuong 1989). These tests compare the log-likelihoods of increasingly complex, or nested, models to ones of simpler models (starting with intercept-only models) and determine the significance of the deviance between the log-likelihoods using a *χ*^2^ test. When significant differences between treatment levels were found, a *post hoc* Tukey's honestly significant difference (HSD) test was applied to the linear predictors using the R package multcomp (Hothorn *et al*. 2008) to detect significant pairwise differences between treatments.

## Results

### Flower contribution to prey attraction

**Figure 3.**
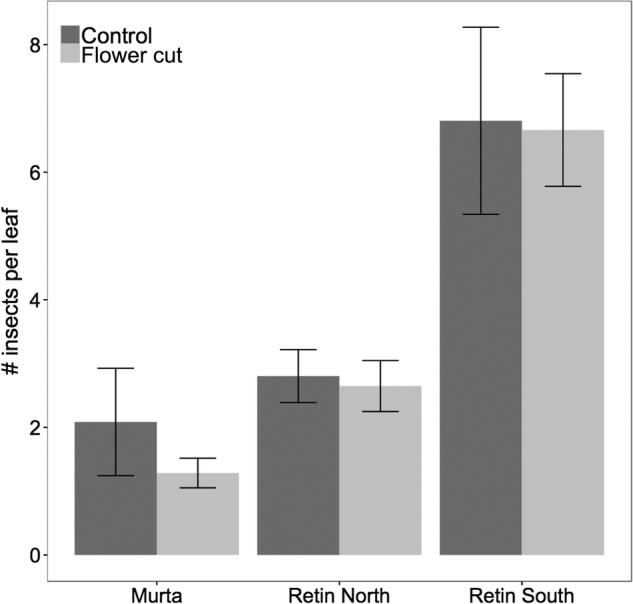
Average number of insects per leaf (±SE) at three sites (Monte Murta, Monte Retin North and Monte Retin South) caught by seven intact flowering plants (control; dark grey bar) and seven plants whose flowers were removed (flower cut; light grey bar).

Overall, insect capture levels differed between the three sites, being considerably higher in Monte Retin South (Fig. 3). However, we detected no significant differences in insect capture rates between ‘flower-removed’ plants and control plants across the three sites (Table 2).

**Table 2.** Results of the likelihood ratio tests for all considered models testing the role of *Drosophyllum* flowers in attracting insects as prey. The response variable (number of insects/leaf) was measured in a field experiment performed at three sites. For each response, a likelihood ratio test compares nested models assuming a chi-square distribution, *χ*^2^, with the critical value given by the model deviance, *D*, and the degrees of freedom, df, corresponding to the difference in parameters between the models compared.

Site	Model	df	*χ*^2^	*D*	*P*
Murta	Intercept		206.9		
	Flower cut	1	204.7	2.2	0.14
Retin North	Intercept		97.6		
	Flower cut	1	95.8	1.9	0.17
Retin South	Intercept		544.7		
	Flower cut	1	541.8	2.7	0.11

### Pollination experiment

Flowers had an overall smaller size (i.e. petal length) in *Drosophyllum* plants from Montera del Torero (average petal length ± SD: 2.64 ± 0.89 cm) than in those from Sierra Carbonera (2.98 ± 0.59 cm; Welch's *t*-test: *t*_64.57_ = 6.46, *P* < 0.0001).

**Figure 4.**
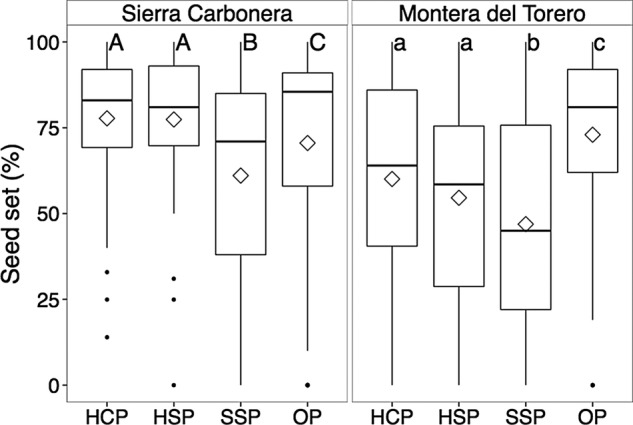
Boxplots of seed set of *D. lusitanicum* after HCP, HSP, SSP and control, OP across two experimental sites (Sierra Carbonera and Montera del Torero). Different letters represent significant pairwise differences (Tukey's HSD, *P* < 0.05) of group means between the four pollination treatments at each site.

Fruit set was very high in *Drosophyllum*, with no differences across the four treatments in the two sites (Table 3) and almost 100 % flowers developing into fruits (Table 4). In contrast, we detected significant differences in seed set among treatments in the two study sites (Table 3). These significant differences were due to the OP treatment, which produced significantly higher seed set than the other three treatments in Montera del Torero (but not in Sierra Carbonera; Table 4; Fig. 4), and particularly the SSP treatment, which produced significantly lower seed set values than the other three treatments at both sites as determined by the HSD test (Table 4; Fig. 4). Seeds were larger and heavier in Sierra Carbonera than in Montera del Torero (Table 4). However, while seeds from the OP treatment in Montera del Torero produced slightly but significantly smaller seeds, no differences in seed size nor weight were detected among treatments in Sierra Carbonera (Tables 3 and 4).

**Table 3.** Results of the likelihood ratio tests for all considered models testing the role of *Drosophyllum* flowers in attracting insects as pollinators. The response variables (fruit set, seed set, seed size and seed weight) were measured in a field experiment performed at two sites (Sierra Carbonera and Montera del Torero). For each response, a likelihood ratio test compares nested models assuming a chi-square distribution, *χ*^2^, with the critical value given by the model deviance, *D*, and the degrees of freedom, df, corresponding to the difference in parameters between the models compared. Significant differences between models are in bold.

Site	Model	df	*χ*^2^	*D*	*P*
Response variable: fruit set					
Sierra Carbonera	Intercept		24.6		
	Pollination	3	22.6	2	0.58
Montera del Torero	Intercept		13.2		
	Pollination	3	11.8	1.4	0.71
Response variable: seed set					
Sierra Carbonera	Intercept		2817.8		
	**Pollination**	**3**	**2706.6**	**140.4**	**<0.01**
Montera del Torero	Intercept		2156.7		
	**Pollination**	**3**	**1958.4**	**198.3**	**<0.01**
Response variable: seed size					
Sierra Carbonera	Intercept		279.3		
	Pollination	3	278.5	0.7	0.9
Montera del Torero	Intercept		291.5		
	**Pollination**	**3**	**282.3**	**9.1**	**0.03**
Response variable: seed weight					
Sierra Carbonera	Intercept		306.4		
	Pollination	3	305.4	1.0	0.8
Montera del Torero	Intercept		128.7		
	**Pollination**	**3**	**110.6**	**18.1**	**<0.01**

**Table 4.** Fecundity variables (fruit set, seed set, seed weight and seed length; mean ± SD) of *D. lusitanicum* per treatment in the two sites. Pairwise significant differences (*P* < 0.05; Tukey's HSD tests) between treatments are indicated by different superscript letters. HCP, hand cross-pollination; HSP, hand self-pollination; SSP, spontaneous self-pollination; OP, control, open pollination.

Treatment	No. of flowers	Fruit set (%)	Seed set (%)	Seed weight (mg)	Seed length (mm)
Sierra Carbonera					
HCP	67	98.5 (±12.2)	77.7 (±18.9)^A^	4.36 (±0.35)	2.48 (±0.13)
HSP	36	100 (±0.0)	77.4 (±22.6)^A^	4.40 (±0.31)	2.48 (±0.15)
SSP	167	99.4 (±7.7)	61.0 (±30.7)^B^	4.35 (±0.45)	2.50 (±0.16)
OP	76	100 (±0.0)	70.6 (±29.7)^C^	4.39 (±0.41)	2.49 (±0.19)
Montera del Torero					
HCP	43	100 (±0.0)	60.0 (±29.1)^a^	3.29 (±0.32)^a^	2.15 (±0.13)^a^
HSP	24	100 (±0.0)	54.6 (±28.2)^a^	3.28 (±0.23)^a^	2.15 (±0.12)^a^
SSP	135	99.3 (±8.6)	47.0 (±31.5)^b^	3.38 (±0.37)^b^	2.15 (±0.17)^a^
OP	65	100 (±0.0)	73.0 (±25.8)^c^	3.16 (±0.31)^a^	2.10 (±0.13)^b^

## Discussion

Although there are no closely related extant species to *Drosophyllum* for comparison (Heubl *et al*. 2006), its large, bright yellow flowers seem to contradict the paradigm of dramatic flower size reduction in highly autogamous angiosperms (Goodwillie *et al*. 2010; Sicard and Lenhard 2011). Considering the presumably high allocation costs of flower production (e.g. Galen 1999; Andersson 2005), we have explored the advantages or benefits that large, conspicuous flowers confer on this highly autogamous, carnivorous plant species.

Since small Diptera (flies) are the main prey insects in *Drosophyllum* (Bertol *et al*. 2015), and the yellow colour is particularly attractive to flies (Neuenschwander 1982; Yee 2015), we tested the hypothesis that large, showy flowers might not be directly related to reproduction, but would instead support plant growth by enhancing prey capture. An increase in prey capture might cause an increase in seed production, as it has been reported in *Drosera* species (Thum 1988), and would therefore have indirect benefits on the reproductive output. However, insect capture rates between intact blooming plants and those plants whose flowers were removed did not differ in any of the three populations (Fig. 3), so we rejected the role of large yellow flowers as significant contributors to prey attraction in *Drosophyllum*.

Considering that the *Drosophyllum* population at Montera del Torero was dominated by old reproductive plants while most reproductive individuals in Sierra Carbonera were young (Table 1), the differences in flower size between the populations can be explained as an allometric effect of plant age. Branching (i.e. number of rosettes) in this species increases with age (Ortega-Olivencia *et al*. 1995; Garrido *et al*. 2003), and flower (or inflorescence) size is known to decrease with branching (Midgley and Bond 1989).

Regarding the controlled pollination experiments, fruit set was very high, with nearly 100 % of the flowers developing into fruit in the four treatments at the two sites (Table 2). Therefore, our results concur with those of Ortega-Olivencia *et al*. (1995, 1998), suggesting that *Drosophyllum* is a highly autogamous species regardless of geographic isolation and population size (Garrido *et al*. 2003; Paniw *et al*. 2015). However, when looking at seed production, some interesting patterns emerged. First, seeds were overall smaller in size and weight in plants from Montera del Torero than in those from Sierra Carbonera (Table 2). Again, this can be attributed to an allometric effect derived from plant age (see above), as there is a strong direct relationship between petal size and seed size in angiosperms (Primack 1987). The slightly but significantly smaller and lighter seeds from the OP treatment in Montera del Torero (Table 2) might be due to the existence of a trade-off between seed number per fruit and seed size/weight (e.g. Baker *et al*. 1994).

Second, while seed set values after the two hand-pollination treatments (HCP and HSP) were remarkably high in Sierra Carbonera, significantly higher than after control, OP, they were significantly lower than after OP in Montera del Torero (Fig. 4). These differences could also be explained by the overall large differences in plant age between reproductive plants of the two populations (Table 1). Since most reproductive plants from Montera del Torero were old, their siring ability might be low, as pollen viability in plants decreases with ageing (Aizen and Rovere 1995; Marshall *et al*. 2010). As only a single anther brush was applied to stigmas of flowers in both HCP and HSP hand-pollination treatments, this could have been sufficient in Sierra Carbonera, where all reproductive plants were young, but not in Montera del Torero. However, we cannot discard differences in weather conditions between populations during the pollination experiments that might have produced different bagging effects. All the same, the lack of differences in seed set between both HCP and HSP treatments in the two populations confirms that no mechanism of self-incompatibility is operating in this species (Ortega-Olivencia *et al*. 1998).

But the most remarkable result found in this study has been the significantly lower seed set values in the SSP treatment at both sites (Table 2; Fig. 4). This means that, even though *Drosophyllum* flowers are readily able to self-pollinate spontaneously, as Ortega-Olivencia *et al*. (1995) had already reported, insect visitation significantly increases seed production by 15−25 % in this species, either by cross-assisted or by insect-assisted self-pollination (facilitated selfing sensu Lloyd 1992). Considering the relatively high rates of seed set after SSP (Ortega-Olivencia *et al*. 1995; this study), may a 15–25 % increase in seed set through insect-assisted pollination offset the costs associated with maintaining large, showy flowers in this highly autogamous species? Its life history and population dynamics suggest an affirmative answer. Adult individuals of this early-successional pyrophyte cannot persist in mature, dense vegetation stands (Paniw *et al*. 2015), whose germination and growth are largely confined to a short post-fire window (Correia and Freitas 2002; M. Paniw, P. Quintana-Ascencio, F. Ojeda and R. Salguero-Gómez, unpublished). In this short temporal window, producing seeds to replenish the seed bank is critical for *Drosophyllum*, as it happens in other pyrophytes (Quintana-Ascencio *et al*. 2003; Menges and Quintana-Ascencio 2004). Therefore, any increase in seed set over autonomous selfing caused by insect visitation—either by facilitated selfing (Lloyd 1992) or by favouring some outcrossing—would increase plant fitness. This, in turn, would account for the maintenance of large, conspicuous flowers in this highly autogamous plant species.

## Conclusions

Although *Drosophyllum* flowers are certainly homogamous (Fig. 1C; Ortega-Olivencia *et al*. 1995), their relatively large, bright yellow corollas challenge the paradigm of autogamous flowers being characterized by a dramatic reduction in corolla size and showiness (Goodwillie *et al*. 2010; Sicard and Lenhard 2011). We rejected the possible role of these flowers as attracting devices for enhancing insect prey capture in this carnivorous species. On the other hand, since the key life-history strategy of this early colonizing pyrophyte is to produce a large, persistent seed bank to maximize post-fire germination (Correia and Freitas 2002; M. Paniw, P. Quintana-Ascencio, F. Ojeda and R. Salguero-Gómez, unpublished), any investment into increasing seed production would have a positive fitness effect. This would thus account for the maintenance of large, showy flowers in a highly autogamous plant species.

## Sources of Funding

This study was supported by the Spanish Ministerio de Economía y Competitividad (project BREATHAL; Geographical barrier, habitat fragmentation and vulnerability of endemics: Biodiversity patterns of the Mediterranean heathland across the Strait of Gibraltar, CGL2011-28759).

## Contributions by the Authors

F.O. conceived the study; F.O., M.P. and R.C.-S. designed the experiments and M.P. A.S.-C. and R.C.-S. carried them out; M.P. and A.S.-C. analysed the data; all authors contributed to writing the manuscript.

## Conflict of Interest Statement

None declared.
